# Supplementary material for: Effects of Trichoderma harzianum combined with Phanerochaete chrysosporium on lignin degradation and humification during chicken manure and rice husk composting
Source: Front Microbiol. 2025 Feb 28;16:1515931. doi: 10.3389/fmicb.2025.1515931 (PMC11906335; doi:10.3389/fmicb.2025.1515931)
Supplement: Supplementary file 1 [file Data_Sheet_1.zip › 6.Function/CK0_BFS/FunGuild/l1/metastats/Saprotroph.CK0_BFS.metastats.pdf]

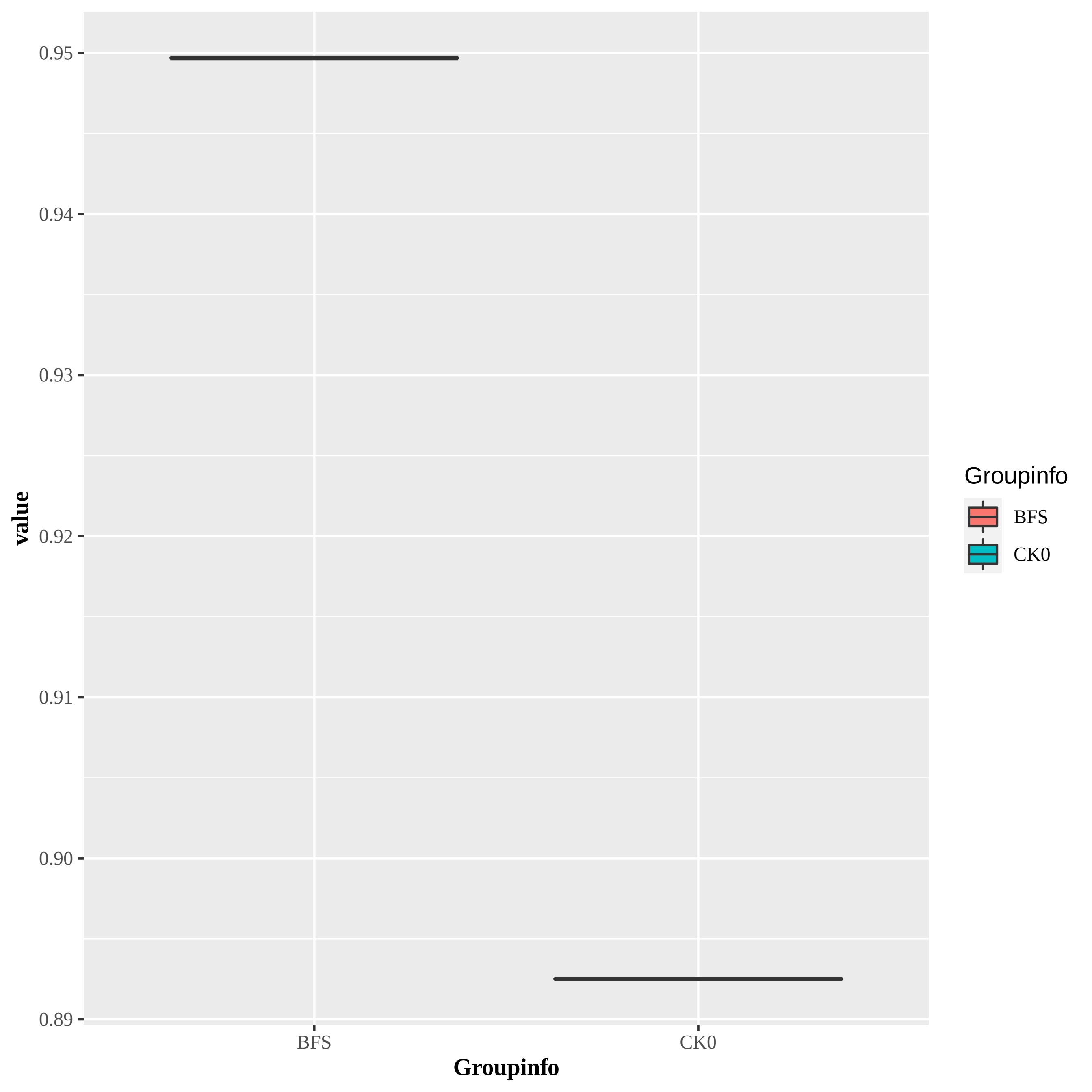
0.95
0.94
0.93
0.92
0.91
0.90
0.89
value
BFS
CK0
Groupinfo
Groupinfo
BFS
CK0
